# Clinicopathological significance of *CHFR* methylation in non-small cell lung cancer: a systematic review and meta-analysis

**DOI:** 10.18632/oncotarget.21962

**Published:** 2017-10-23

**Authors:** Chen Wang, Wenxia Ma, Rong Wei, Xiaoqin Zhang, Ningning Shen, Lifang Shang, Li E, Ying Wang, Lifang Gao, Xin Li, Bin Wang, Yaping Zhang, Aiping Du

**Affiliations:** ^1^ Department of Pathology, The Second Hospital of Shanxi Medical University, Taiyuan, Shanxi, 030001, P.R. China

**Keywords:** CHFR, NSCLC, biomarker, methylation, ADC

## Abstract

Checkpoint with Forkhead-associated and Ring finger domains (*CHFR*) is a G2/M checkpoint and tumor-suppressor gene. Recent publications showed the correlation of *CHFR* promoter methylation with clinicopathological significance of non-small cell lung cancer (NSCLC), however, the results remain inconsistent. The aim of this study is to investigate the Clinicopathological significance of *CHFR* promoter methylation in NSCLC with a meta-analysis. A total of nine studies were included in the meta-analysis that 816 patients were involved. Our data indicated that the frequency of *CHFR* promoter methylation was higher in NSCLC than in normal lung tissue, Odd Ratios (OR) was 9.92 with 95% corresponding confidence interval (CI) 2.17–45.23, *p* = 0.003. Further subgroup analysis revealed that *CHFR* promoter was more frequently methylated in squamous cell carcinoma (SCC) than in adenocarcinoma (ADC), OR was 4.46 with 95% CI 1.65–12.05, *p* = 0.003, suggesting the mechanism of SCC pathogenesis is different from ADC. Notably, *CHFR* promoter methylation was correlated with smoking behavior in NSCLC. In conclusion, *CHFR* could be a biomarker for diagnosis of NSCLC, and a promising drug target for development of gene therapy in SCC. *CHFR* promoter methylation is potentially associated with poor overall survival, additional studies need to be carried out for confirmation in future.

## INTRODUCTION

Lung cancer is one of the most common malignancies and the leading cause of cancer-related mortality in the world. Lung cancer can be classified into two major histological groups, small cell lung cancer and non-small cell lung cancer (NSCLC). NSCLC accounts for more than 80% of all lung cancers, whereas 15–20% is small cell lung cancer [1, 2]. NSCLC can be divided into three subtypes of adenocarcinoma (ADC), squamous cell carcinoma (SCC) and large-cell carcinoma, within them, adenocarcinomas accounts for 40%, squamous cell carcinoma for 25–30%, and large-cell carcinoma for 10–15% [3, 4]. Although lung cancer subtypes share some genetic variations such as inactivation of tumor suppressor gene *TP53*, each subtype harbors its own specific genetic variations such as *c-MET* in ADC, *fibroblast growth factor receptor 1* (*FGFR1*) and *FGFR3* in SCC.

DNA methylation is a part of the epigenetic gene regulation complex and plays a critical role in carcinogenesis [5]. Recently, specific molecular alterations that drive tumor growth and provide targets for therapy have been defined in ADC, but there is increasing interest in the molecular landscape of SCC highlighting new potential therapeutic targets [6]. Checkpoint with Forkhead-associated and Ring finger domains (*CHFR*) is a G2/M checkpoint gene that has been identified by Scolnik and Halazonetis [7]. This protein contains a forkhead and a RING finger domain, and functions as an ubiquitin ligase that ubiquitinates target proteins to direct them to the proteasome for degradation or to alter their activity [8, 9]. The growing evidence supports its role as a tumor-suppressor protein and biomarker for chemotherapeutic response to microtubule-targeting drugs such as taxanes [9]. *CHFR* promoter hypermethylation has been observed in several tumors such as 30% in esophageal cancer [10], 20% in NSCLC [11] and 40% in colorectal cancer (CRC) [12]. However, the frequency of *CHFR* hypermethylation in NSCLC was inconsistent, and its contribution to the development and progression of NSCLC are unclear due to the small power of individual study. Therefore we pooled nine studies and performed a meta-analysis to evaluate the clinicopathologic significance of *CHFR* hypermethylation in NSCLC.

## RESULTS

### Identification of relevant studies and quality assessment

**Figure 1:**
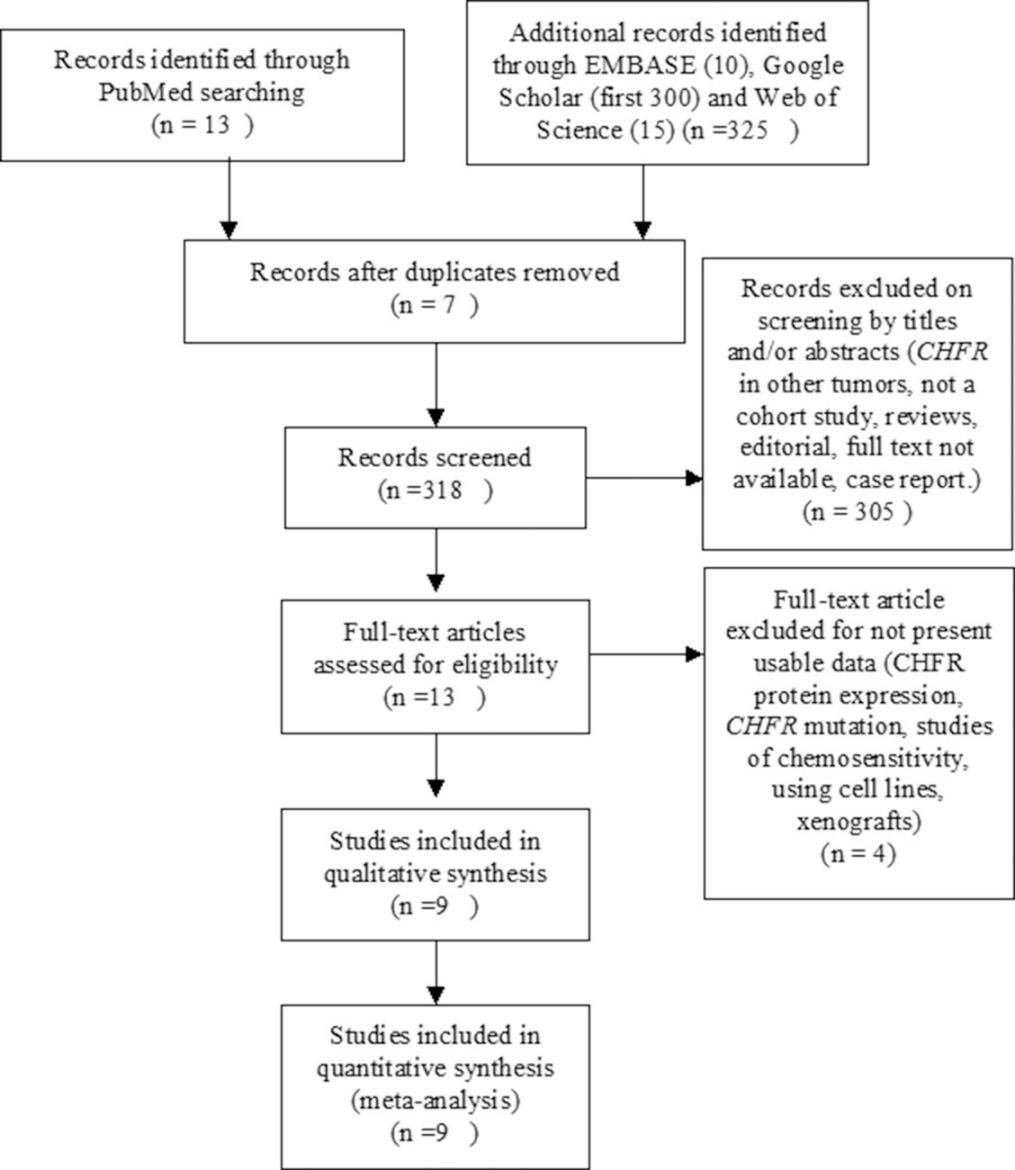
Schematic flow diagram for selection of included studies

A total of nine studies were included in the meta-analysis (Figure 1) and 816 participants from five countries were involved. The study characteristics was summarized in Table 1. Based on the quality evaluation with the Newcastle-Ottawa Quality Assessment Scale (NOQAS), the overall quality of nine studies was scored from six to eight which indicated good quality (data not shown).

**Table 1:** Main characteristics of included studies

Author	Year	Country	Sample size (NSCLC)	Histology			Stage (TNM)		Grade		Smoking status		Method
				NCT	ADC	SCC	I+II	III+IV	L	H	+	-	
Guo [22]	2015	USA	20/195	1/100	7/101	13/94	11/100	2/25	-	-	-	-	MSP
Pillai [42]	2013	Japan	1/32	-	-	-	-	-	-	-	-	-	MSP
Koga [21]	2011	Japan	28/205	-	16/165	12/40	26/183	3/226	4/71	25/135	23/126	6/82	MSP
Salazar [43]	2011	Spain	32/91	-	-	-	-	-	-	-	-	-	MSP
Takeshita [20]	2010	Japan	11/68	-	2/48	9/20	10/56	1/12	-	-	11/48	0/21	MSP
De Jong [44]	2009	Belgium	2/10	0/18	-	-	-	-	-	-	-	-	MSP
Takeshita[45]	2008	Japan	3/20	-	-	-	-	-	-	-	-	-	MSP
Corn [39]	2003	USA	2/20	0/20	-	-	-	-	-	-	-	-	MSP
Mizuno [11]	2002	Japan	7/37	-	-	-	-	-	-	-	-	-	MSP

MSP: methylation-specific PCR, NCT: normal control tissue; AC: Adenocarcinoma; SCC: Squamous Cell Cancer; L: low grade; H: high grade;

### The frequency of *CHFR* promoter methylation in AC and SCC, and the association with the status of smoking

**Figure 2:**
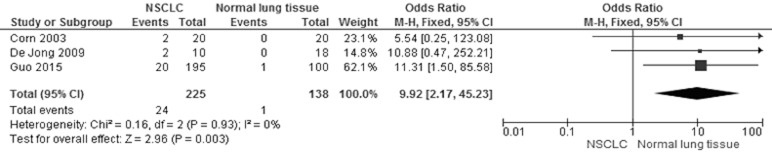
Forest plot for *CHFR* promoter methylation in NSCLC and normal lung tissue The squares represent the weight of individual study in the meta-analysis, the line width indicates the corresponding 95% CI, The diamond represents the pooled OR, and the width of diamond indicates 95% CI.

**Figure 3:**
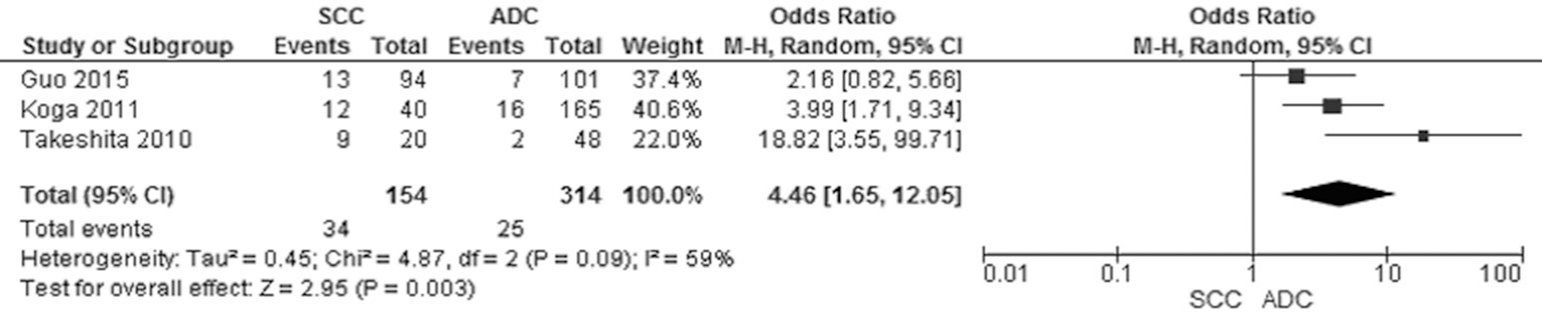
Forest plot for *CHFR* promoter methylation in SCC and ADC The squares represent the weight of individual study in the meta-analysis, the line width indicates the corresponding 95% CI, The diamond represents the pooled OR, and the width of diamond indicates 95% CI.

**Figure 4:**
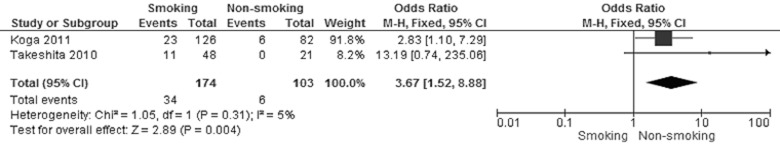
Forest plot for *CHFR* promoter methylation in NSCLC patients with smoking and non-smoking behavior The squares represent the weight of individual study in the meta-analysis, the line width indicates the corresponding 95% CI, The diamond represents the pooled OR, and the width of diamond indicates 95% CI.

Of the total 678 NSCLC patients from nine studies, aberrant *CHFR* promoter methylation was identified in 106 patients, the frequency was 15.6%. Three studies reported the comparison of *CHFR* promoter methylation in NSCLC and normal lung tissue, the pooled rate of *CHFR* promoter methylation was significantly higher in NSCLC than normal lung tissue, OR was 9.92, 95% CI 2.17–45.23, test for overall effect, *Z* = 2. 96, *p* = 0.003 (Figure 2). Three studies investigated *CHFR* promoter methylation in a total of 314 AC and 154 SCC respectively, *CHFR* promoter methylation in 34 out of 154 SCC (22.1%) and 25 out of 314 AC (8.0%) were identified. The frequency of *CHFR* promoter methylation was significantly higher in SCC than AC, OR was 4.46, 95% CI 1.65–12.05, test for overall effect, *Z* = 2. 95, *p* = 0.003 (Figure 3). Two studies investigated the relationship between *CHFR* promoter methylation in NSCLC and the status of smoking, pooled data indicated that *CHFR* promoter was more frequently methylated in smoking NSCLC patients than in non-smoking patients, OR was 3.67 with 95% CI 1.52–8.88, test for overall effect, Z = 2. 89, *p* = 0.004 (Figure 4).

### The association between *CHFR* promoter methylation and NSCLC stages as well as prognosis

**Figure 5:**
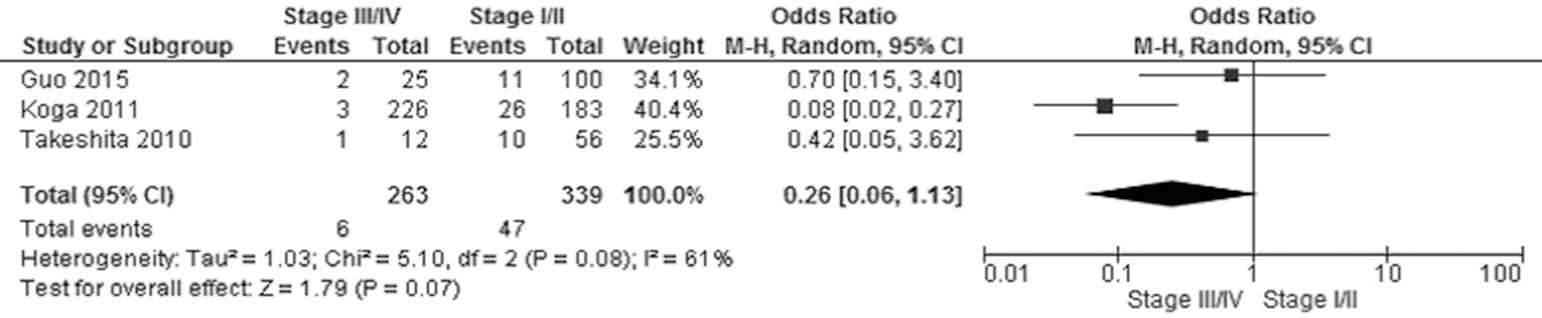
Forest plot for *CHFR* promoter methylation in NSCLC stage III/IV and stage I/II The squares represent the weight of individual study in the meta-analysis, the line width indicates the corresponding 95% CI, The diamond represents the pooled OR, and the width of diamond indicates 95% CI.

*CHFR* promoter methylation was not associated with NSCLC stages, the frequency of *CHFR* promoter methylation in stage III/IV NSCLC was not significantly increased compared to stage I/II NSCLC, OR was 0.26, 95% CI 0.06–1.13, test for overall effect, *Z* = 1.79, *p* = 0.007 (Figure 5). Two studies showed *CHFR* promoter methylation or low CHFR expression was correlated with poor prognosis, respectively. (Table 2).

**Table 2:** The association between CHFR status and overall survival (HR) in NSCLC patients

Author	Sample Size	CHFR	HR	*P* value	Treatment
Koga [21]	208	Unmethylation vs. Methylation	3.44 (1.15–10.29)	0.0274	Surgery
Takeshita [45]	157	Nuclear stain High vs. Low	3.260 (1.189–8.938)	0.021	Surgery

### Sensitivity analysis and publication bias

**Figure 6:**
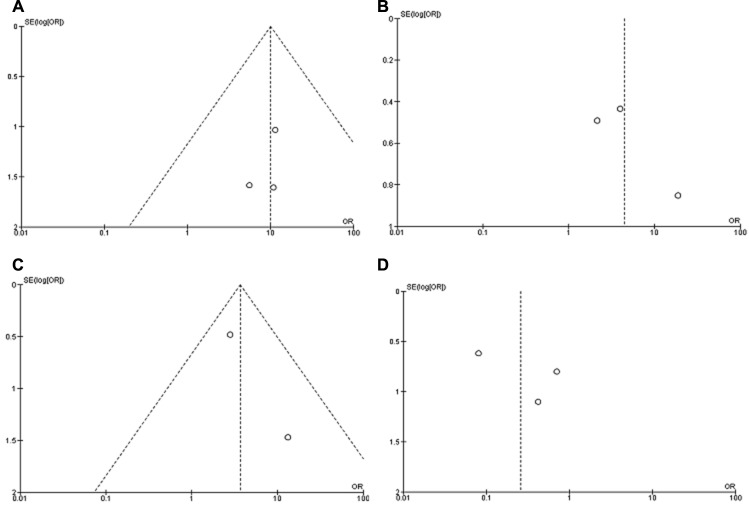
Funnel plot for publication bias (**A**) *CHFR* promoter methylation in NSCLC and normal lung tissue; (**B**) *CHFR* promoter methylation in SCC and ADC; (**C**) *CHFR* promoter methylation in NSCLC patients with smoking and non-smoking behavior; (**D**) *CHFR* promoter methylation in NSCLC stage III/IV and stage I/II. S.E., standard error; Area of the circle represents the weight of individual study.

A sensitivity analysis was performed by removing one study at a time, the ORs were not significantly changed, indicating the stability of present meta-analysis (Supplementary Figure 1, Supplementary Figure 2, Supplementary Figure 3, Supplementary Figure 4). The funnel plots were largely symmetric (Figure 6), suggesting there was no publication biases existed in the meta-analysis of relationship between *CHFR* promoter methylation and clinicopathological characteristics.

## DISCUSSION

Scolnik and Halazonetis were the first to report the lack of *CHFR* gene in colorectal cancer and neuroblastoma cell lines, after then loss of CHFR expression has been observed in a variety of malignancies such as colorectal cancer [12–14], gastric cancer [15–18], esophageal cancer [10, 19] and NSCLC [20–22]. Previous evidence indicated *CHFR* was mostly inactivated by its promotor CpG island methylation [23]. *CHFR* promoter methylation has been observed in NSCLC, however, the frequency varied from 3.1% to 35.1% due to small size of samples. We pooled nine studies together and calculated the frequency of *CHFR* promoter methylation in 678 NSCLC patients, it was 15.6%. Three studies evaluated the rate of *CHFR* promoter methylation in NSCLC and normal lung tissue, pooled OR suggested *CHFR* promoter methylation in NSCLC was ten times higher than in normal lung tissue. Previous evidence indicated that CHFR interacts with beta-tubulin and TCTP (Translationally Controlled Tumor-associated *Protein*) to stabilize microtubule [24]. Disruption of spindle cause CHFR deliberate from TCTP and spindle, this process results in the activation of signal pathway and delay cell cycle progression [25]. CHFR regulates the coordination of chromosome condensation and centrosome separation during prophase [7]. Thus, loss of CHFR causes errors in chromosome segregation that can lead to neoplasia [9]. Additional studies indicated that CHFR ubiquitinates and targets both polo-like-kinase (PLK1) and Aurora A, leads to the inhibition of phosphorylation of cell division cycle 25 (Cdc25), which in turn control the Cdc2 kinase activity at G2 to M transition [26–30]. Ultimately, the cyclin B1-Cdk complex is not able to form and the cell cycle is arrested [29, 31]. Therefore, the cells with *CHFR* gene inactivated by promoter methylation cannot be arrested in the G2 phase and proceed to mitosis, and proliferation and abnormal differentiation leads to the development of NSCLC and its progression.

Further subgroup analysis revealed that *CHFR* promoter was more frequently methylated in SCC than ADC, OR was 4.46 with 95% CI 1.65–12.05, *p* = 0.003 (Figure 3), suggesting that inactivation of *CHFR* gene was associated with the development of SCC. The molecular mechanism of pathogenesis is probably different between SCC and ADC. In addition, prior studies demonstrated that the loss of several suppressor genes such as *Wnt inhibitory factor-1* (*Wif1*) [32], *Phosphatase and tensin homolog deleted on chromosome 10* (*PTEN*) [33] and *TP53* [34–36] occurred more frequently in SSC than in ADC. P53 mutations were the most common one in SCC, occurring at 50% of cases, however there was no relationship between CHFR expression and p53 mutation [37], indicating CHFR may contribute the carcinogenesis of SCC independently. Interestingly, present data showed that *CHFR* promoter methylation was associated with smoking behavior which is a risk factor for the development of SSC [38]. Similarly, previous reports indicated that alterations of *Wif1, PTEN* and *TP53* gene were associated with smoking behavior [32–35], suggesting that smoking behavior may lead to the squamous cell carcinogenesis by inducing the inactivation of those suppressor genes including *CHFR* gene.

Unlike ADC, there are no target therapies used in treatment of SCC patients, therefore, *CHFR* could be a potential drug target for development of gene therapies in SCC via demethylation. Notably, in cancer cells with methylated *CHFR*, treatment with demethylation agent 5-aza-2-deoxycytidine led to re-expression of CHFR, and partially restored the prophase checkpoint [39]. Moreover demethylation agents such as azacitidine (AZA) has been demonstrated in reversing the effects of hypermethylation in solid tumors [40]. Thus, CHFR could be a very promising drug target for personalized treatment in patients with SCC.

The frequency of *CHFR* promoter methylation was higher in stage I/II of NSCLC than in stage III/IV, showing that *CHFR* methylation occurred at early stage during the development of NSCLC. Additional analysis needs to be carried out when more relevant studies are available. *CHFR* promoter methylation was potentially associated with the overall survival based on the two included studies, although further studies will be needed to broadly establish this association in NSCLC.

The limitations of this meta-analysis are as follows, first, present findings were based on individual unadjusted ORs, and further confirmation needs to be finished by other potential risk factors. The second, publication bias may exist, as positive results were more likely published. The third, there are some clinical and statistical heterogeneity between the included studies. The fourth, most included studies are from Japan, therefore, the finding of the meta-analysis should be interpreted with caution.

In conclusion, *CHFR* promoter methylation is correlated with the risk of SSC development. *CHFR* could be a potential biomarker and drug target to develop personalized treatment for the patients with SCC. *CHFR* promoter methylation is associated with smoking behavior.

## MATERIALS AND METHODS

### Study identification

Searches were performed from the earliest available data to July 2017 in PubMed, EMBASE, Web of Science and google scholar. The search terms were “non-small cell lung cancer”, “NSCLC”, “methylation”, and “*CHFR*, or Checkpoint with Forkhead-associated and Ring finger domains”. There were 13 articles identified from PubMed, 15 articles from Web Science, 10 articles from Embase. 1940 articles were identified from Google scholar, first 300 of them were reviewed since the rest of them are not related to the present study. The reference lists of Included studies were checked for any further relevant citations.

The inclusion criteria consisted of the following: 1). Articles evaluated methylation of *CHFR* in NSCLC; 2). Articles studied the relationship between *CHFR* methylation and clinicopathological features in NSCLC. Exclusion criteria were studies using cell line and human xenografts, as well as using the same population and overlapping database. The flow chart of searches is shown in Figure 1.

### Data extraction

Primary data were extracted by using a customized form which included first author, year of publication, geography location, methylation methods, histology categories of NSCLC, stages, grades and status of smoking. Two reviewers extracted the data independently, any disagreements were discussed until a consensus was reached.

### Quality assessment

The methodological quality of included studies was evaluated using NOQAS. This scale was used to allocate a maximum of nine points, 0–4 points for selection, 0–2 points for comparability, 0–3 points for outcomes. The NOS scores ranged from 0 to 9, and a score ≥ 7 indicates a good quality. All studies were rated by two reviewers independently, any disagreements were discussed until a consensus was reached.

### Statistical analysis

The pooled ORs with its 95% confidence intervals were calculated. The heterogeneity among studies was determined by using the Cochran’s Q statistic and *I*^2^ tests. When the *I*^2^ value was below 50%, fixed effect model was used, when the *I*^2^ value was 50% or greater, a random effect model was used. Publication bias was assessed by using a method reported by Egger et al. [41]. The meta-analysis was performed using Review Manager 5.3 (Cochrane Collaboration, Software Update, Oxford, UK). *P*-value less than 0.05 was considered statistically significant.

## SUPPLEMENTARY MATERIALS FIGURES


