# Supplementary material for: Clinicopathological significance of CHFR methylation in non-small cell lung cancer: a systematic review and meta-analysis
Source: Oncotarget. 2017 Oct 23;8(65):109732–9. doi: 10.18632/oncotarget.21962 (PMC5752556; doi:10.18632/oncotarget.21962)
Supplement: Supplementary file 1 [file oncotarget-08-109732-s001.pdf]

Clinicalpathological significance of *CHFR* methylation in non-small cell lung cancer: a systematic review and meta-analysis

SUPPLEMENTARY MATERIALS

Study or Subgroup	NSCLC Events	NSCLC Total	Normal lung tissue Events	Normal lung tissue Total	Weight	Odds Ratio M-H, Fixed, 95% CI
Corn 2003	2	20	0	20	23.1%	5.54 [0.25, 123.08]
De Jong 2009	2	10	0	18	14.8%	10.88 [0.47, 252.21]
Guo 2015	20	195	1	100	62.1%	11.31 [1.50, 85.58]
**Total (95% CI)**		**225**		**138**	**100.0%**	**9.92 [2.17, 45.23]**
Total events	24		1			

Heterogeneity: Chi^2 = 0.16, df = 2 (P = 0.93); I^2 = 0%
Test for overall effect: Z = 2.96 (P = 0.003)

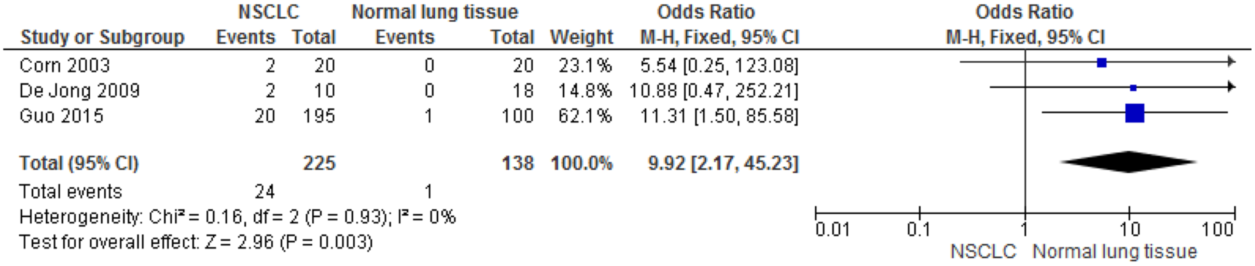

Study or Subgroup	NSCLC Events	NSCLC Total	Normal lung tissue Events	Normal lung tissue Total	Weight	Odds Ratio M-H, Fixed, 95% CI
De Jong 2009	2	10	0	18	19.3%	10.88 [0.47, 252.21]
Guo 2015	20	195	1	100	80.7%	11.31 [1.50, 85.58]
**Total (95% CI)**		**205**		**118**	**100.0%**	**11.23 [1.97, 64.18]**
Total events	22		1			

Heterogeneity: Chi^2 = 0.00, df = 1 (P = 0.98); I^2 = 0%
Test for overall effect: Z = 2.72 (P = 0.007)

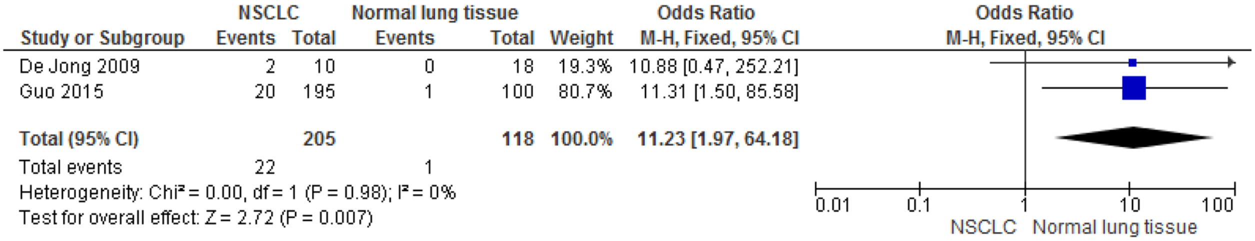

Study or Subgroup	NSCLC Events	NSCLC Total	Normal lung tissue Events	Normal lung tissue Total	Weight	Odds Ratio M-H, Fixed, 95% CI
Corn 2003	2	20	0	20	27.1%	5.54 [0.25, 123.08]
Guo 2015	20	195	1	100	72.9%	11.31 [1.50, 85.58]
**Total (95% CI)**		**215**		**120**	**100.0%**	**9.75 [1.79, 53.27]**
Total events	22		1			

Heterogeneity: Chi^2 = 0.15, df = 1 (P = 0.70); I^2 = 0%
Test for overall effect: Z = 2.63 (P = 0.009)

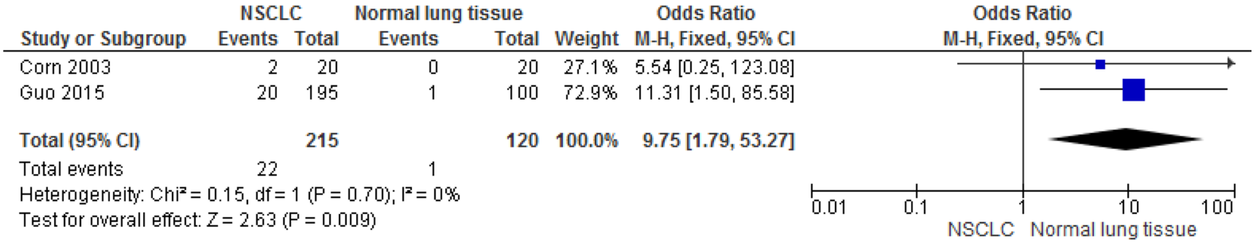

Study or Subgroup	NSCLC Events	NSCLC Total	Normal lung tissue Events	Normal lung tissue Total	Weight	Odds Ratio M-H, Fixed, 95% CI
Corn 2003	2	20	0	20	60.9%	5.54 [0.25, 123.08]
De Jong 2009	2	10	0	18	39.1%	10.88 [0.47, 252.21]
**Total (95% CI)**		**30**		**38**	**100.0%**	**7.63 [0.83, 69.76]**
Total events	4		0			

Heterogeneity: Chi^2 = 0.09, df = 1 (P = 0.76); I^2 = 0%
Test for overall effect: Z = 1.80 (P = 0.07)

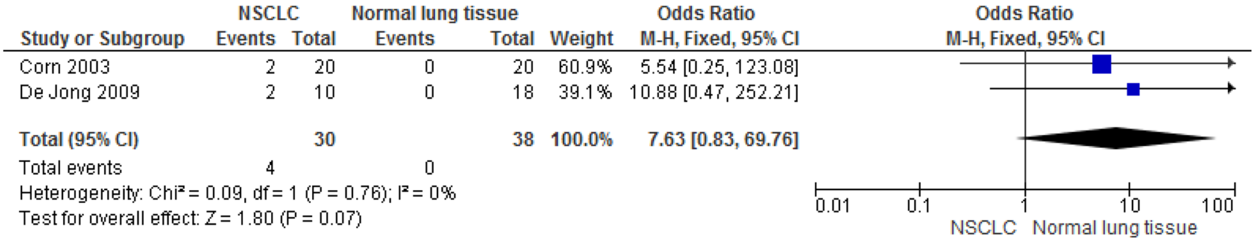

Supplementary Figure 1: Sensitive analysis for *CHFR* promoter methylation in NSCLC and normal lung tissue.

Study or Subgroup	SCC Events	SCC Total	ADC Events	ADC Total	Weight	Odds Ratio M-H, Random, 95% CI
Guo 2015	13	94	7	101	37.4%	2.16 [0.82, 5.66]
Koga 2011	12	40	16	165	40.6%	3.99 [1.71, 9.34]
Takeshita 2010	9	20	2	48	22.0%	18.82 [3.55, 99.71]
**Total (95% CI)**		**154**		**314**	**100.0%**	**4.46 [1.65, 12.05]**
Total events	34		25			

Heterogeneity: Tau² = 0.45; Chi² = 4.87, df = 2 (P = 0.09); I² = 59%
Test for overall effect: Z = 2.95 (P = 0.003)

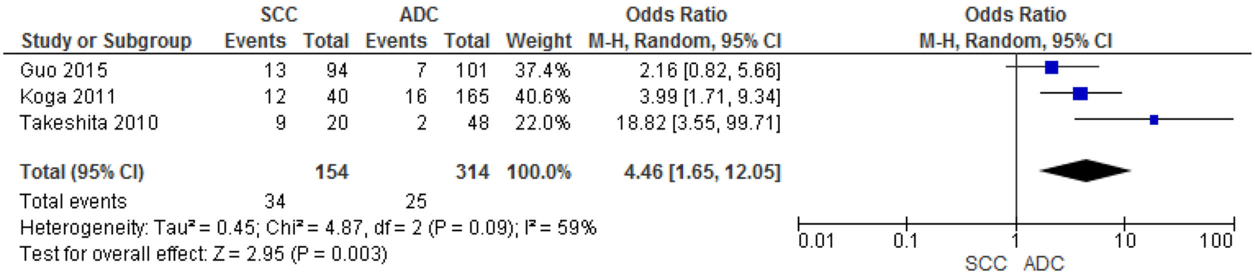

Study or Subgroup	SCC Events	SCC Total	ADC Events	ADC Total	Weight	Odds Ratio M-H, Random, 95% CI
Koga 2011	12	40	16	165	61.0%	3.99 [1.71, 9.34]
Takeshita 2010	9	20	2	48	39.0%	18.82 [3.55, 99.71]
**Total (95% CI)**		**60**		**213**	**100.0%**	**7.31 [1.64, 32.49]**
Total events	21		18			

Heterogeneity: Tau² = 0.76; Chi² = 2.67, df = 1 (P = 0.10); I² = 63%
Test for overall effect: Z = 2.61 (P = 0.009)

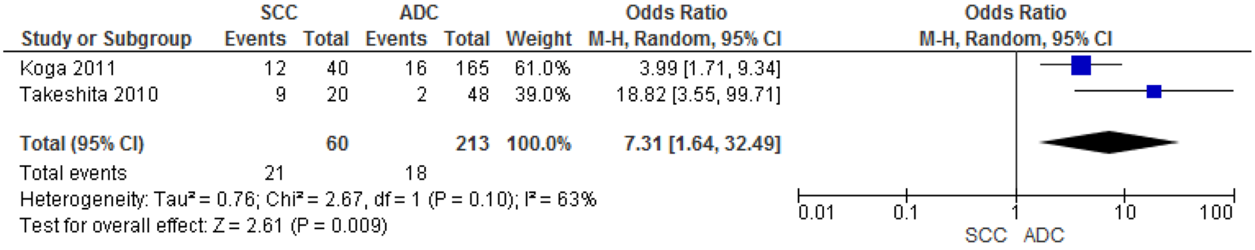

Study or Subgroup	SCC Events	SCC Total	ADC Events	ADC Total	Weight	Odds Ratio M-H, Random, 95% CI
Guo 2015	13	94	7	101	55.1%	2.16 [0.82, 5.66]
Takeshita 2010	9	20	2	48	44.9%	18.82 [3.55, 99.71]
**Total (95% CI)**		**114**		**149**	**100.0%**	**5.70 [0.69, 47.18]**
Total events	22		9			

Heterogeneity: Tau² = 1.87; Chi² = 4.86, df = 1 (P = 0.03); I² = 79%
Test for overall effect: Z = 1.61 (P = 0.11)

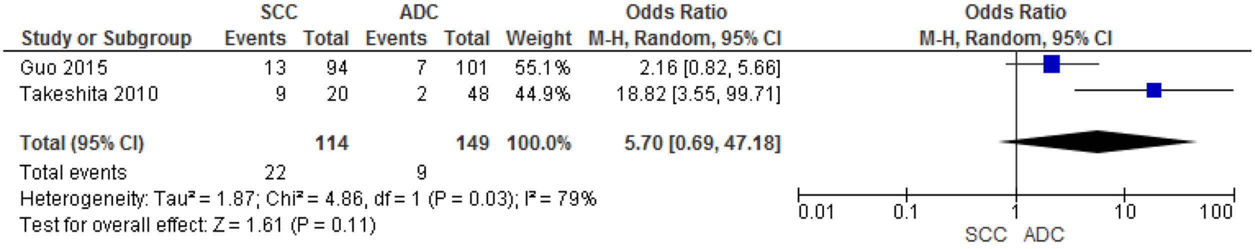

Study or Subgroup	SCC Events	SCC Total	ADC Events	ADC Total	Weight	Odds Ratio M-H, Random, 95% CI
Guo 2015	13	94	7	101	43.7%	2.16 [0.82, 5.66]
Koga 2011	12	40	16	165	56.3%	3.99 [1.71, 9.34]
**Total (95% CI)**		**134**		**266**	**100.0%**	**3.05 [1.61, 5.77]**
Total events	25		23			

Heterogeneity: Tau² = 0.00; Chi² = 0.89, df = 1 (P = 0.34); I² = 0%
Test for overall effect: Z = 3.42 (P = 0.0006)

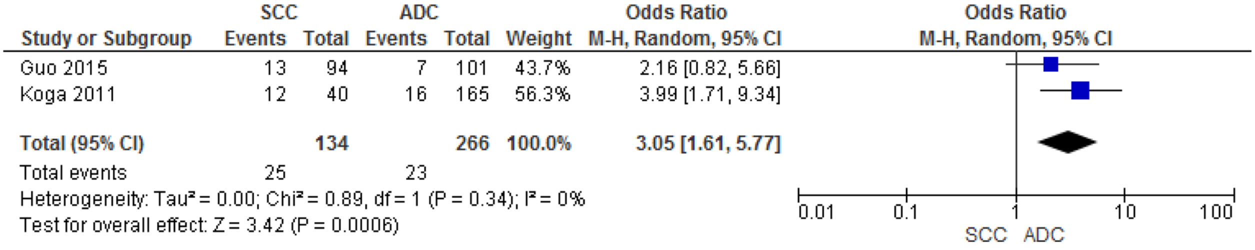

Supplementary Figure 2: Sensitive analysis for *CHFR* promoter methylation in SCC and ADC.

Study or Subgroup	Smoking Events	Smoking Total	Non-smoking Events	Non-smoking Total	Weight	Odds Ratio M-H, Fixed, 95% CI
Koga 2011	23	126	6	82	91.8%	2.83 [1.10, 7.29]
Takeshita 2010	11	48	0	21	8.2%	13.19 [0.74, 235.06]
**Total (95% CI)**		**174**		**103**	**100.0%**	**3.67 [1.52, 8.88]**
Total events	34		6			

Heterogeneity: Chi² = 1.05, df = 1 (P = 0.31); I² = 5%

Test for overall effect: Z = 2.89 (P = 0.004)

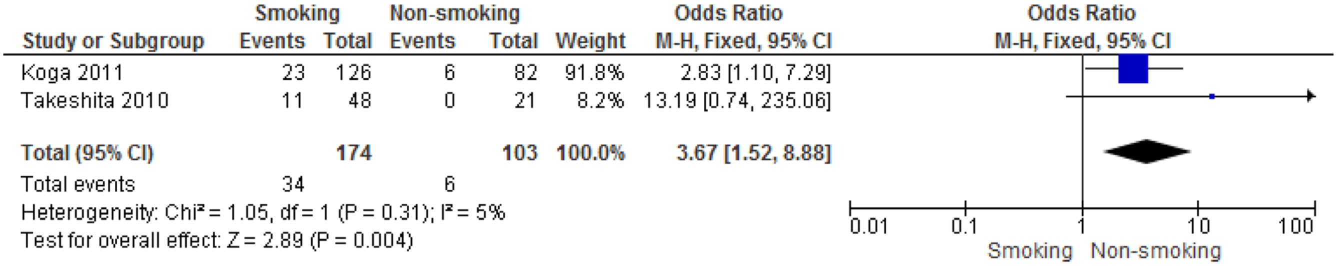

Study or Subgroup	Smoking Events	Smoking Total	Non-smoking Events	Non-smoking Total	Weight	Odds Ratio M-H, Fixed, 95% CI
Takeshita 2010	11	48	0	21	100.0%	13.19 [0.74, 235.06]
**Total (95% CI)**		**48**		**21**	**100.0%**	**13.19 [0.74, 235.06]**
Total events	11		0			

Heterogeneity: Not applicable

Test for overall effect: Z = 1.75 (P = 0.08)

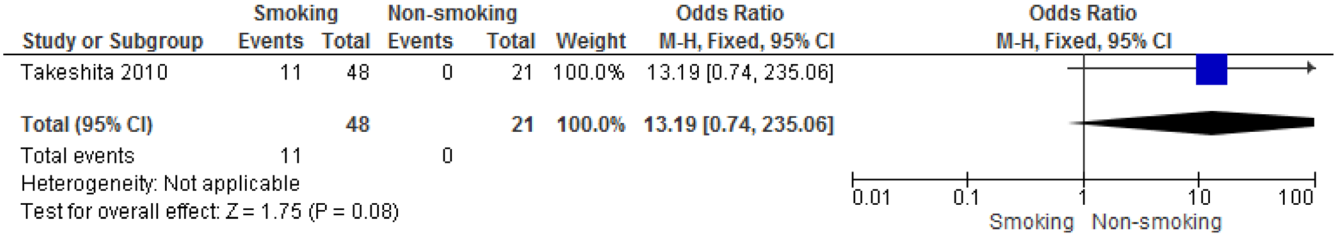

Study or Subgroup	Smoking Events	Smoking Total	Non-smoking Events	Non-smoking Total	Weight	Odds Ratio M-H, Fixed, 95% CI
Koga 2011	23	126	6	82	100.0%	2.83 [1.10, 7.29]
**Total (95% CI)**		**126**		**82**	**100.0%**	**2.83 [1.10, 7.29]**
Total events	23		6			

Heterogeneity: Not applicable

Test for overall effect: Z = 2.15 (P = 0.03)

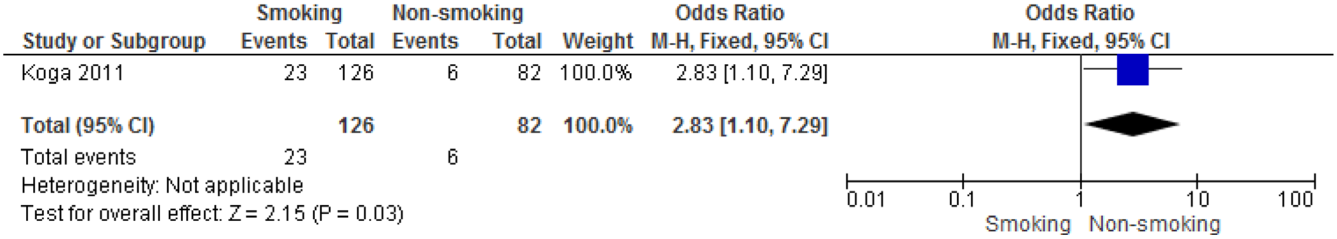

Supplementary Figure 3: Sensitive analysis for *CHFR* promoter methylation in NSCLC patients with smoking and non-smoking behavior.

	Stage III/IV		Stage I/II			Odds Ratio
**Study or Subgroup**	**Events**	**Total**	**Events**	**Total**	**Weight**	**M-H, Random, 95% CI**
Guo 2015	2	25	11	100	34.1%	0.70 [0.15, 3.40]
Koga 2011	3	226	26	183	40.4%	0.08 [0.02, 0.27]
Takeshita 2010	1	12	10	56	25.5%	0.42 [0.05, 3.62]
**Total (95% CI)**		**263**		**339**	**100.0%**	**0.26 [0.06, 1.13]**
Total events	6		47			

Heterogeneity: Tau² = 1.03; Chi² = 5.10, df = 2 (P = 0.08); I² = 61%
Test for overall effect: Z = 1.79 (P = 0.07)

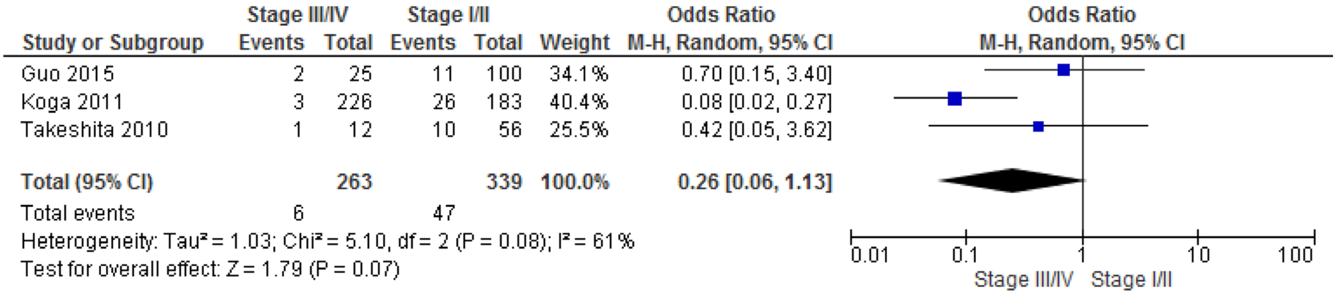

	Stage III/IV		Stage I/II			Odds Ratio
**Study or Subgroup**	**Events**	**Total**	**Events**	**Total**	**Weight**	**M-H, Random, 95% CI**
Koga 2011	3	226	26	183	65.4%	0.08 [0.02, 0.27]
Takeshita 2010	1	12	10	56	34.6%	0.42 [0.05, 3.62]
**Total (95% CI)**		**238**		**239**	**100.0%**	**0.14 [0.03, 0.66]**
Total events	4		36			

Heterogeneity: Tau² = 0.55; Chi² = 1.69, df = 1 (P = 0.19); I² = 41%
Test for overall effect: Z = 2.49 (P = 0.01)

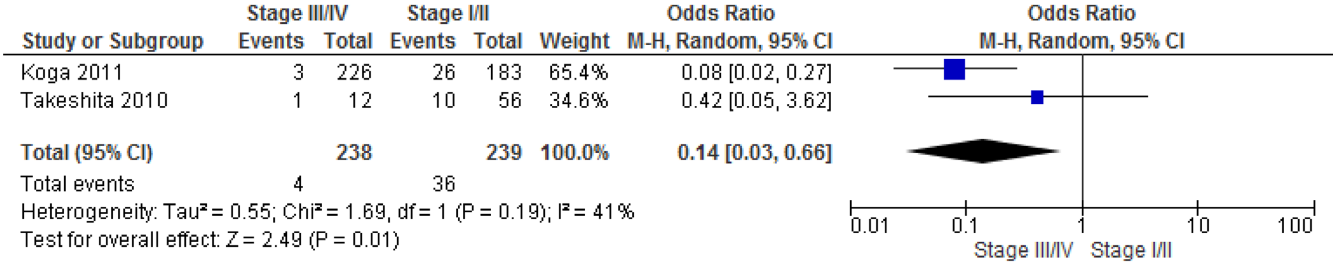

	Stage III/IV		Stage I/II			Odds Ratio
**Study or Subgroup**	**Events**	**Total**	**Events**	**Total**	**Weight**	**M-H, Random, 95% CI**
Guo 2015	2	25	11	100	65.3%	0.70 [0.15, 3.40]
Takeshita 2010	1	12	10	56	34.7%	0.42 [0.05, 3.62]
**Total (95% CI)**		**37**		**156**	**100.0%**	**0.59 [0.16, 2.10]**
Total events	3		21			

Heterogeneity: Tau² = 0.00; Chi² = 0.15, df = 1 (P = 0.70); I² = 0%
Test for overall effect: Z = 0.82 (P = 0.41)

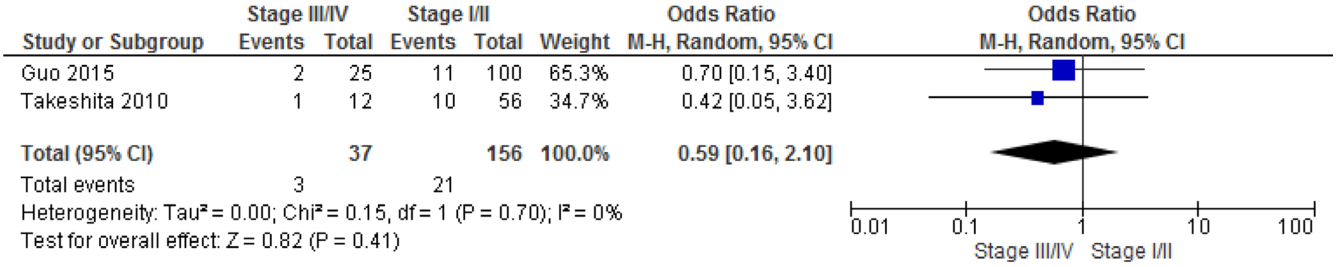

	Stage III/IV		Stage I/II			Odds Ratio
**Study or Subgroup**	**Events**	**Total**	**Events**	**Total**	**Weight**	**M-H, Random, 95% CI**
Guo 2015	2	25	11	100	47.2%	0.70 [0.15, 3.40]
Koga 2011	3	226	26	183	52.8%	0.08 [0.02, 0.27]
**Total (95% CI)**		**251**		**283**	**100.0%**	**0.23 [0.03, 1.89]**
Total events	5		37			

Heterogeneity: Tau² = 1.85; Chi² = 4.60, df = 1 (P = 0.03); I² = 78%
Test for overall effect: Z = 1.37 (P = 0.17)

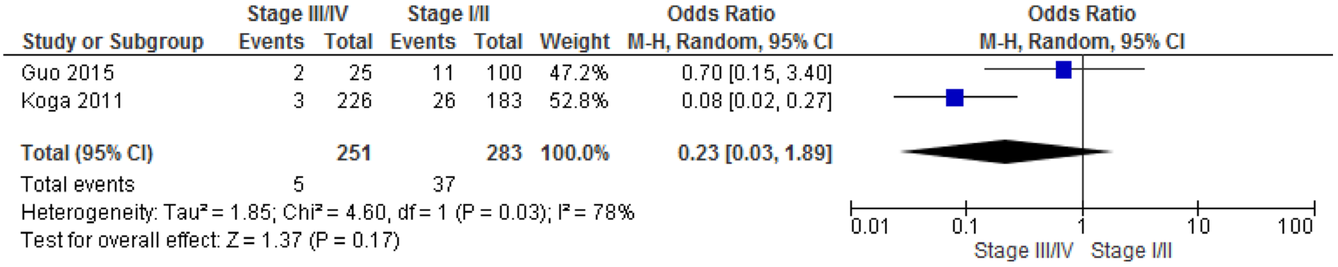

Supplementary Figure 4: Sensitive analysis for *CHFR* promoter methylation in NSCLC stage III/IV and stage I/II.
